# Understanding Changes in Violent Extremist Attitudes During the Transition to Early Adulthood

**DOI:** 10.1007/s10940-021-09522-9

**Published:** 2021-07-08

**Authors:** Amy Nivette, Lea Echelmeyer, Frank Weerman, Manuel Eisner, Denis Ribeaud

**Affiliations:** 1 Department of Sociology, Utrecht University, Padualaan 14, 584 CH Utrecht, The Netherlands; 2 Netherlands Institute for the Study of Crime and Law Enforcement (NSCR), Amsterdam, The Netherlands; 3 Faculty of Law, Vrije Universiteit, Amsterdam, The Netherlands; 4 Erasmus School of Law, Erasmus University, Rotterdam, The Netherlands; 5 Institute of Criminology, University of Cambridge, Cambridge, UK; 6 Jacobs Center for Productive Youth Development, University of Zurich, Zürich, Switzerland

**Keywords:** Violent extremist attitudes, Radicalization, Deradicalization, Maturation, Strain

## Abstract

**Objectives:**

The current study seeks to explain changes in support for violent extremism during the transition to early adulthood. This period during the life course could increase uncertainty and vulnerability to radicalization, or alternatively lead to maturation, prosocial bonds, and consequently less support for violent extremism. In the absence of population-based longitudinal data on violent extremist attitudes, we know very little about how and why attitudes change during this period.

**Method:**

Data came from an ongoing longitudinal cohort study in Zürich, Switzerland (n = 910). First, we assessed the variation in violent extremist attitudes between ages 17 and 20 using the Reliable Change Index. Second, we used hybrid regression techniques to investigate to what extent theoretically relevant factors can explain between- and within-individual differences in violent extremist attitudes.

**Results:**

Our results show that violent extremist attitudes are largely stable or declining between late adolescence and early adulthood, and that within-individual changes in low self-control, conflict coping skills, and peer disapproval of violence can in part explain these changes.

**Conclusions:**

For young people in Zürich, the transition to early adulthood was characterized by increases in psychosocial maturity, more prosocial peers, and less deviant behavior, which in turn was associated with lower support for violent extremism. Existing research on effective interventions for criminal desistance and disengagement from gangs may therefore be fruitful avenues for developing programs aimed at reducing support for violent extremism and fostering deradicalization.

**Supplementary Information:**

The online version contains supplementary material available at 10.1007/s10940-021-09522-9.

## Introduction

The transition between adolescence and early adulthood is particularly important for understanding processes of radicalization and changes in violent extremist attitudes (Jahnke et al. 2020; Schils and Verhage 2017). On the one hand, violent extremist attitudes may increase as identity-seeking youths are more vulnerable to new and potentially radicalizing peers, media, and other social environments (Bizina and Gray 2014; Bouhana and Wikström 2010; Hapvarkin 2019; Jahnke et al. 2020). On the other hand, support for violent extremism may decrease as youth develop stronger ties to adult social institutions of control, adopt more conventional values, and reach psychosocial maturity (Forrest and Hay 2011; Sampson and Laub 1993; Steinberg et al. 2015; Warr 1998). To date, we know very little about how violent extremist attitudes change over the life course, and to what extent these life course transitions and maturation processes contribute to increases and decreases in violent extremist thinking (Wolfowicz et al. 2019).

This limitation is largely due to the lack of prospective longitudinal information on violent extremist attitudes (see for reviews, Lösel et al. 2018; Wolfowicz et al. 2019). While models of radicalization outline factors that explain within-individual changes in attitudes (e.g., ‘stepwise’ models, Moghaddam 2005), studies are often only able to assess between-individual differences using cross-sectional data (Curran and Bauer 2011). Evidence for between-individual relations is useful for identifying characteristics of individuals who support violent extremism, but does not necessarily imply that changes in these risk factors are associated with changes in support. In relation to criminal behavior, there can be significant discrepancies between within- and between-individual effects (Farrington et al. 2002; Keijsers 2016; Rekkers et al. 2017).

In light of these limitations, this study has two goals: first, to assess patterns of individual change in violent extremist attitudes between late adolescence and early adulthood. In particular, we explore to what extent attitudes are stable, increasing, or decreasing during this period of transition. Second, drawing from theoretical models of radicalization and desistance, we examine whether and which social and individual characteristics can account for between- and within-individual variations in violent extremist attitudes. In order to do so, we utilize data from the Zürich Project on Social Development from Childhood to Adulthood (z-proso), an ongoing prospective longitudinal survey of an ethnically and religiously diverse sample of youth in Zürich, Switzerland.

To our knowledge, the current study is one of the very few that contains prospectively measured support for violent extremism over time. In addition, we were able to assess a wide array of theoretically-relevant experiential, social, and psychological factors in relation to both between- and within-individual differences in violent extremist attitudes. Support for violent extremism was measured at two time points during the transition from adolescence to adulthood (ages 17 and 20), meaning that the data are ideal for exploring different developmental processes that can contribute to radicalization or deradicalization among youth.

### Background: Defining and Framing Violent Extremism, Radicalization and Deradicalization

Before we turn to our literature review, we want to clarify our primary perspective on the central concepts of this study. We define violent extremist attitudes as beliefs that justify or endorse violence to achieve social, political, religious, or ideological goals (Borum 2011; International Association of Chiefs of Police 2014). Radicalization refers to the gradual process by which individuals increasingly acquire and develop violent extremist attitudes (Doosje et al. 2016; McCauley and Moskalenko 2008). Deradicalization by contrast refers to the process by which individuals abandon their extremist ideology and support for extremist violence (Braddock 2014; Koehler 2020). In theoretical models of radicalization, support for violent extremism is typically considered an important early precursor to engagement in extremist groups and acts (Borum 2011; McCauley and Moskalenko 2008; Wolfowicz et al. 2019). However, it is important to acknowledge that the vast majority of individuals who support violent extremism do not progress to commit violence themselves (Wikström and Bouhana 2016). Reviews of research indicate that violent extremist attitudes are a moderately strong correlate of violent extremist behaviors, but the available research suggests there are likely different mechanisms contributing to the development of attitudes compared to behaviors (Wolfowicz et al. 2019). This paper therefore focuses on the mechanisms and processes that influence the development of violent extremist attitudes.

Importantly, in this study we focus on *general* support for violent extremism to achieve different political, ideological, religious, or economic goals. While previous research has identified notable differences in correlates of religious, right-, and left-wing extremist violence (Chermak and Gruenewald 2015; LaFree et al. 2018), extremist ideologies share an underlying endorsement for the use of violence to achieve these goals. In addition, a recent meta-analysis found that the effects of risk and protective factors on radical attitudes did not substantially differ between ideologically-mixed and ideologically-specific samples (Wolfowicz et al. 2019). By examining the factors that contribute to general support for extremist violence, we can better identify any general mechanisms and processes that can be targeted through early prevention.

In recent years, there has been increased attention to the integration and application of criminological theories and knowledge to understanding processes leading to violent extremist attitudes (Decker and Pyrooz 2015; Della Porta and LaFree 2012; Freilich and LaFree 2015; LaFree et al. 2018). Systematic reviews show that ‘traditional’ criminological risk factors derived from theories such as general strain theory, social learning theory, and social bond theory show relatively strong associations with violent extremist attitudes across studies (Lösel et al. 2018; Wolfowicz et al. 2019). There have been several attempts to integrate these different approaches into a comprehensive theoretical framework of radicalization and violent extremism (e.g. Chermak and Gruenewald 2015; Pauwels and De Waele 2014; Wikström and Bouhana 2017; Van den Bos 2019). These integrated approaches are broadly reflective of a social-ecological framework for explaining violence over the life course, which assumes that violence is shaped by the interplay of causal processes at the individual, the relational, the community, and the societal levels (Bronfenbrenner 1979; Krug et al. 2002). Within this framework, individuals are seen as being part of ongoing transactional (i.e. bi-directional) dynamics, whereby they are both exposed to environments while at the same time also actively shaping their social environments. A life course perspective further proposes that changes must be understood within the social–historical context of a given life stage (Schulenberg and Schoon 2012; Shanahan 2000). In the current study, this means that we must view these experiential, social, and individual influences within the context of the transition to adulthood. During this stage, young people may be transitioning between educational roles or into occupational roles and forming new social networks and intimate relationships (Eccles et al. 2003; Lageson and Uggen 2013; Swisher and Dennison 2016; Young and Rees, 2013) while continuing their own cognitive and social development (McCuish et al. 2020). Negative life events that occur during this stage, such as break-ups, failure to enter the labor market, or serious victimization, can disrupt the timing and success of transition into adult roles, inhibiting or delaying the adoption of adult roles and responsibilities (Eccles et al. 2003; MacMillan and Hagan 2004). Building on these socio-ecological and life course frameworks, we therefore consider three broad domains that reflect theoretical mechanisms that are expected to increase the risk of radicalization: negative life experiences, peer influences, and individual attitudes and propensities.

## Theoretical Framework

### Radicalization and Strains

An important distal factor that can influence motivations to adopt violent extremist beliefs is an individual’s exposure to negative life experiences (Agnew 2000; Kruglanski et al. 2019). According to General Strain Theory [GST], negative life experiences are forms of strain that can produce negative emotions such as anger that demand corrective action (Agnew 1993). These adverse experiences can include discrimination or injustice, job loss, romantic breakup, victimization or abuse, or negative encounters with authorities (Kalamakis and Chandler 2015). Criminal behavior is seen as a form of corrective action that seeks to injure or damage the presumed source of strain. Within this perspective, violent extremism is most likely to emerge as corrective action in response to collective strains, such as exposure to trauma from war or group discrimination (Agnew 2010; Nivette et al. 2017).

Significance quest theory highlights a range of potential individual and social (collective) experiences or events that can increase the likelihood of supporting violent extremism by activating a quest for significance (e.g. Jasko et al. 2017). Significance quest theory (Kruglanski et al. 2014, 2019) posits that negative life experiences can activate feelings of humiliation, loss of control, and uncertainty that motivate people to adopt more extreme ideologies and behaviors in order to regain a sense of self-worth and personal significance (Jasko et al. 2017). Violent extremist beliefs offer a particularly salient narrative for “lost” individuals, as they provide an opportunity to restore significance as well as the tools to seek revenge on the source of humiliation or injustice (Kruglanski et al. 2019).

Experiences of social exclusion and marginalization are considered particularly important sources for significance quests leading to radicalization (Bäck et al. 2018; Lyons-Padilla et al. 2015; Pretus et al. 2018). Social exclusion reflects processes by which individuals or groups are disconnected or “shut out” from conventional society (Foster and Hagan 2007). Social exclusion can be understood as a “rupture of the social bond” with society (Silver 1994, quoted in Foster and Hagan, p. 400), introducing adverse social and psychological conditions such as isolation, uncertainty, feelings of unfairness, and the need to belong, which in turn increase vulnerability to deviant and extremist values (Bäck et al. 2018; van den Bos 2019, 2020).

Research in criminology suggests that individuals are more likely to commit crime during periods in which they experienced strain (within-individual effects), such as stressful life events or violent victimization (Felson et al. 2012; Hoffman 2019; Slocum et al. 2005). Studies have shown that exposure to both individual and collective strains are associated with higher support for violent extremism between individuals (Pauwels and De Waele 2014; Pauwels and Schils 2017; Nivette et al. 2017; Simi et al. 2016; Wolfowicz et al. 2019). However, there are currently no longitudinal studies that have evaluated within-individual effects of strain on violent extremist attitudes.

Youth may be particularly susceptible to negative life experiences during late adolescence, with serious consequences for establishing stable adult roles and social supports (MacMillan and Hagan 2004). In addition, adolescents are more sensitive to injustice (Janke et al. 2020) and not yet adept to cope with adverse experiences compared to adults (Agnew 2003; Hoffman 2010; Jensen et al. 2016).

While some argue that experiences of collective strain are more important to motivate violent extremism (Agnew 2010; Nivette et al. 2017; Pauwels and De Waele 2014), both GST and significance quest theory highlight a range of negative life experiences that are expected to contribute to negative emotions and/or feelings of uncertainty (Hogg et al. 2013; Jasko et al. 2017). In theory as well as the media, a wide range of personal negative life events occurring prior to radicalization are often presented as the “trigger” motivating radicalization or violent extremist acts (e.g., job loss, academic failure, victimization, divorce, see Kruglanski et al. 2014). The current study therefore focuses on the role of personal strains in motivating the adoption of violent extremist attitudes.

Based on GST and significance quest theory, we expect that youths who experience overall higher exposure to strain will have stronger support for violent extremist attitudes (between-individual effects). Drawing from evidence on GST and crime, we also expect that during periods in which youth experience strain/negative life experiences, they will have higher support for violent extremism (within-individual effects).

### Radicalization and Peers

From a social learning perspective, peers play an important role in providing justifications and situational stimulation for rule-breaking and extremist violence (Pauwels and De Waele 2014; Wikström and Bouhana 2017), as well as access to extremist groups or settings (Simi et al. 2016). According to Akers and Silverman (2004, p. 27), “terrorists learn an ideology that the ends justify the means; violence for political ends is accepted and rewarded. These function as definitions favorable to violence.” In other words, association with peers that espouse values and beliefs favorable to violence increases the probability that individuals similarly adopt such beliefs, which in turn can motivate violent extremist action given the opportunity (Pauwels and Schils 2016). Even individual actors, or so-called “loners”, can learn tactics and adopt justifications for extremist violence through online networks (Holt et al. 2019). In addition, youth with higher levels of support for violent extremism may also seek out like-minded peers (Botha 2014).

While there is evidence that individuals who associate with deviant or extremist peers have on average higher support for violent extremism (e.g. Pauwels et al. 2018; Wolfowicz et al. 2019), there is a lack of evidence regarding within-individual effects. However, research from criminology suggests that individuals are more likely to engage in criminal behavior during periods in which they were exposed to deviant peers (Beardslee et al. 2018; Osgood et al. 1996; Weerman 2011). We therefore expect that association with deviant peers can explain both between- and within-individual variations in violent extremist attitudes.

### Radicalization and Individual Characteristics

Research has highlighted a number of individual characteristics that reflect an individual’s susceptibility and propensity to adopt violent extremist attitudes (see e.g., Lösel et al. 2018; Wolfowicz et al. 2019). These factors are considered more proximal since they reflect more immediate influences on values and behaviors (Pauwels and De Waele 2014). We focus in particular on four characteristics that have been shown to be important to predicting violent extremist attitudes or violence more generally: low self-control, previous deviant behavior, competent conflict coping strategies, perceived police legitimacy.

Dispositional characteristics such as low self-control reflect latent tendencies to engage in rule-breaking and adopt deviant values, including violent extremism (Simi et al. 2016; Wikström and Bouhana 2017; Wolfowicz et al. 2019). Individuals with low self-control are more likely to seek immediate gratification, more risk-taking, impulsive, and self-centered (Gottfredson and Hirschi 1990). Low self-control has proven to be a relatively robust risk factor for criminal and violent behaviors (Pratt and Cullen 2000; Vazsonyi et al. 2017). While Hirschi and Gottfredson (2001) initially theorized that self-control is not relevant for understanding ideologically-motivated offenders because committing terrorist attacks requires long-term planning and foresight, studies have shown that low self-control is a relatively robust risk factor for violent extremist outcomes (Pauwels and De Waele 2014; Pauwels and Svensson 2017; Pauwels et al. 2018; Rottweiler et al. 2021; Wolfowicz et al. 2019). Although conceived as a time-stable trait that accounts for between-individual differences in criminal behavior, there is growing evidence that low self-control is malleable over the life course, and that within-individual changes in low self-control can explain changes in criminal behavior (Burt et al. 2014; Hay et al. 2010; Huijsmans et al. 2019). Forrest and Hay (2011) argue that reductions in criminal behavior during adulthood can in part be explained by improvements in self-control. If we assume that low self-control influences violent extremist attitudes in the same way, we can expect that low self-control is associated with both between- and within-individual differences in violent extremist attitudes among youth.

Past involvement in deviant behavior is also an important indicator of the potential for future deviance and rule-breaking (Assink et al. 2015), and this may be extended to violent extremism. In research on radicalization, criminal history (or conversely, law abidance) has been found to be a relatively strong (between-individual) correlate of violent extremist attitudes and behaviors (LaFree et al. 2018; Lösel et al. 2018; Simi et al. 2016; Wolfowicz et al. 2019). We therefore expect that individuals who report more deviant behavior will have higher support for violent extremism. To our knowledge there are no studies examining within-individual effects of criminal behavior on violent extremist attitudes. If the development of violent extremist attitudes follows the age-crime curve, it is logical to expect that during periods of more engagement in deviant or criminal behavior, individuals will report higher levels of violent extremist attitudes.

Another factor that determines an individual’s susceptibility to radicalization is the extent to which one is able to competently cope with adversity and stress (Agnew 2010; Feddes et al. 2015; Nivette et al. 2017). Competent conflict coping is characterized by emotional self-regulation, positive communication, prosocial relationships, and empathy (Bornstein et al. 2010). From the perspective of both GST and significance quest theory, individuals who are able to competently cope with negative experiences are less likely to experience significance loss or the need for corrective action (Agnew 2006, 2010). Prior studies have found that elements of competent coping, such as perspective-taking and empathy, were negatively related to violent extremist attitudes and behaviors (Lösel et al. 2018; Nivette et al. 2017). Moreover, an evaluation of resilience training among Dutch Muslim adolescents found that increasing perspective-taking and empathy significantly reduced support for ideology-based violence (Feddes et al. 2015). Based on this evidence, we expect that both between-individual differences and within-individual change in competent coping skills are negatively associated with differences in violent extremist attitudes.

The extent to which individuals perceive the police, law, and other legal institutions to be legitimate has been identified as an important factor influencing vulnerability to radicalization (Pauwels et al. 2018; van den Bos 2019). Theoretically, when people perceive the police to treat them with fairness, neutrality, and respect, they are more likely to voluntarily comply with the law (Tyler 2006). Empirical evidence suggests that between-individual differences in perceptions of police legitimacy are associated with a range of criminal and violent outcomes (Walters and Bolger 2019) including violent extremist attitudes (Pauwels and De Waele 2014). However, evidence supporting within-individual effects of police legitimacy on criminal behavior is relatively weak (Augustyn 2015; Kaiser and Reisig 2019; Nagin and Telep 2017). In particular, Kaiser and Reisig (2019) found that perceptions of police legitimacy explained between-individual differences in offending, but not within-individual changes. They argued that while longer-term or overall levels of police legitimacy can help us understand differences in criminal behavior, short-term fluctuations in police legitimacy may not be enough to influence changes in criminal behavior. Based on existing cross-sectional evidence on criminal and violent extremist outcomes, we can expect that police legitimacy will explain between-individual variation in violent extremist attitudes. However, there is no strong evidence to suggest that within-individual changes in police legitimacy will explain changes in violent extremist attitudes.

### Radicalization and Age/Maturation

The transition to early adulthood is typically accompanied by desistance from criminal behavior (Southamer-Loeber et al. 2004). Desistance theories propose that this reduction in criminal behavior occurs due to a series of dynamic social and psychological processes that take place in early adulthood (Moffitt 1993; Rocque 2015; Sampson and Laub 1993; Steinberg et al. 2015). As youth enter into adulthood, they begin to invest in new social roles, responsibilities, and relationships that together work to inhibit criminal activities and promote conventional values (Massoglia and Uggen 2010; Sampson and Laub 1993). At the same time, psychosocial maturation means that youth are better able to control their impulses, resist peer influence, and take the perspective of others (Mulvey et al. 2004; Rocque et al. 2015; Steinberg et al. 2009). Taken together, this suggests that the period between late adolescence and early adulthood may also see significant declines in vulnerability to and support for violent extremism.

Researchers have recently begun to draw parallels between processes contributing to desistance from crime and deradicalization (Bubolz and Simi 2015; Della Porta and LaFree 2012; Decker and Pyrooz 2015). In a recent meta-analysis, Wolfowicz and colleagues (2019) found that age was consistently negatively related to violent extremist attitudes and behaviors, suggesting overall declines over time. Significance quest theory proposes that deradicalization occurs when individuals gain a sense of significance through prosocial means, such as marriage, becoming a parent, or gaining employment (Doosje et al. 2016). These turning points mirror the mechanisms that contribute to criminal desistance and disengagement from gangs (Decker et al. 2014; Sampson and Laub 1993). Studies on deradicalization and disengagement from hate groups show that motivations for change often involve new adult roles and responsibilities in the form of employment, marriage, and children (Bubolz and Simi 2015; Schuurman and Bakker 2016; Sikkens et al. 2017).

Radicalization may therefore follow a similar “age-crime curve” trajectory, where support declines with psychosocial maturation and increasing investment in social roles in adulthood. If violent extremist attitudes follow the “age-crime curve,” we expect that average support will decline between ages 17 and 20.

### The Current Study

In the absence of population-based longitudinal data on violent extremist attitudes, we know very little about how and why these attitudes change over time. The current study aims to fill this important gap in the literature first by examining changes in violent extremist attitudes during the transition from adolescence to early adulthood, and second by analyzing the impact of personal strains and individual characteristics on between- and within-individual variation in these attitudes among a sample of maturing youth in Zurich, Switzerland. To achieve this, we formulated the following research questions:

1. How do violent extremist attitudes change between the ages of 17 and 20, and which patterns of stability, increases and/or decreases can be identified?
2. To what extent can personal strains and negative life experiences explain between and within-individual variation in violent extremist attitudes?
3. To what extent can associations with deviant peers and personal characteristics explain between- and within-individual variation in violent extremist attitudes?

Based on theoretical models on radicalization and the criminological literature on the correlates of radicalization and deradicalization, we can formulate the following expectations about these research questions:

1. We expect that average support for violent extremism will decline between ages 17 and 20.
2. Based on General Strain Theory and significance quest theory, we expect that youth who experience overall higher exposure to strain will have stronger support for violent extremist attitudes (between-individual effects). Drawing from evidence on GST and crime, we also expect that during periods in which youth experience strain / negative life experiences, youth will have higher support for violent extremism (within-individual effects).
3. Based on the criminological literature and systematic reviews on radicalization and deradicalization, we expect that low self-control, previous deviant behavior, poor coping strategies, low perceived police legitimacy and association with deviant peers are related to between-individual variation in violent extremist attitudes, and that levels of self-control, deviant behavior, coping strategies, and association with deviant peers are also related to within-individual changes in these attitudes (but that perceived police legitimacy is not).

## Methods

This study uses data from the Zürich Project on Social Development from Childhood to Adulthood (z-proso), an ongoing prospective longitudinal study on the development of prosocial and problem behaviors. The z-proso data is based on a cohort of children who entered 1 of the 56 primary schools in the city of Zürich in 2004 (see Eisner et al. 2011, for details). The initial target sample of schools was selected using random sampling procedures in which disadvantaged school districts were oversampled. Despite oversampling lower SES school districts, the study population is largely representative of the youth population in Zürich, but not Switzerland more generally. A large proportion of youths have a migration background (45% in the current sample), with the largest proportion of migrant parents born in the former Yugoslavia, Sri Lanka, Portugal, Germany, and Turkey. The study consists of eight waves of interviews at ages 7, 8, 9, 11, 13, 15, 17, and 20 (see Eisner et al. 2019, for information on attrition from waves 1–7). In the first wave, 81 percent of the target sample participated (n = 1360). Active written parental consent was required for the first 6 years of participation. At age 13 and 15, participating youths were legally old enough to give the active consent to participate on their own, while their parents received an information letter that allowed them to proscribe their child’s participation (passive consent procedure).

Violent extremist attitudes were measured at ages 17 and 20. At age 17, 1305 youths participated in the survey, and at age 20, 1180 participated. At age 17, participants completed paper/pencil questionnaires in classrooms outside of regular lesson times. At age 20, participants completed surveys on a computer at a university research laboratory. Participants received ~ US$60 at age 17 and ~ US$75 at age 20 for completing the questionnaire.

In order to assess changes over time, the sample was restricted to those who participated in both waves (n = 1117)^1^ and for whom complete information was available (n = 910). Overall, the percentage of missing data for each variable was low, ranging from 0.08 to 7.8%. An analysis of attrition between waves 5 (age 13) and 7 (age 17) showed that attrition was not related to antisocial and problem behaviors (Eisner et al. 2019). However, between waves 7 (age 17) and 8 (age 20), dropouts are significantly more likely to be male, immigrant, low socio-economic status, and aggressive, but did not show more deviant behavior (see Nivette et al. 2020: 78). Dropouts between ages 17 and 20 also report significantly more support for violent extremist attitudes (M_dropout_ = 1.94 vs. M_age20_ = 1.81, t = 2.39, *p* < 0.01). Implications for the analyses and results are discussed in the limitations section.

### Measures

#### Dependent Variable: Violent Extremist Attitudes Scale

The violent extremist attitude scale was developed by the z-proso team to measure generic support for violent extremism defined as attitudes that condone the use of violence to achieve collective goals (see Nivette et al. 2017: 764–765). The scale consists of four items, with responses measured on a four-point Likert-type scale ranging from “fully untrue” to “fully true”. The items included were as follows:

- “It’s sometimes necessary to use violence to fight against things that are very unjust;”
- “Sometimes people have to resort to violence to defend their values, convictions, or religious beliefs;”
- “It’s OK to support groups that use violence to fight injustices;”
- “It’s sometimes necessary to use violence, commit attacks, or kidnap people to fight for a better world.”

The reliability over time was good, with a Cronbach’s α of 0.80 in both waves.

#### Independent Variables

In accordance with significance quest theory and strain perspectives (Agnew 2006; Kruglanski et al. 2014), and following Jasko et al.’s (2017) operationalization, we selected a range of *adverse life events* most relevant for the transition to adulthood that reflect dimensions of social or relationship loss (i.e. break-up with partner, break-up with friend), failure to achieve aspirations (i.e. school censure, had to repeat a grade, did not find apprenticeship, cancelled training), trauma (i.e. serious victimization, victim of domestic violence), and negative contacts with the criminal justice system (i.e. negative police contact, spent time in custody). For all but the victimization items, respondents were asked at age 17 and 20 whether or not the event happened in the previous 2 years. For victimization, respondents were asked to report whether they were the victim of robbery, assault leading to an injury committed with a weapon, assault leading to an injury committed without a weapon, or serious sexual assault in the 12 months prior to the survey. The responses were used to create a dichotomous variable that indicates whether the respondent had been a victim of violent crime. A complete list of adverse events and their prevalence rates are reported in Table 1. We constructed dichotomous variables reflecting whether the respondent experienced at least one adverse event within each dimension (i.e. social loss, achievement loss, trauma, criminal justice contact).

**Table 1:** Descriptive characteristics for adverse life events representing strain and significance loss

	t1: age 17			t2: age 20		
Adverse life event	N	M/%	SD	N	M/%	SD
*Achievement loss*						
School censure	909	10	0.30	910	13	0.33
Repeated grade	910	6	0.24	910	7	0.26
No apprenticeship	906	13	0.34	909	6	0.23
Cancelled training	906	5	0.22	910	10	0.30
*Relational loss*						
Break-up with partner	907	29	0.46	910	46	0.50
Break-up with friend	909	16	0.36	910	26	0.44
*Trauma*						
Victim of domestic violence	904	1	0.12	910	4	0.19
Violent victimization	910	8	0.26	910	9	0.28
*Contact with CJS*						
Police contact	909	7	0.25	910	10	0.31
Spent time in custody	910	4	0.60	910	1	0.12

SD = standard deviation

*Social Exclusion* was measured using 6 items that capture the respondent’s feelings of segregation, alienation, worthlessness, and lack of opportunities in society (e.g. “I have the feeling that I’m not really part of society”). The items were measured using a four-point Likert-type scale (i.e. from “fully untrue” to “fully true”). The scale was not measured at age 17, so we used the most recent measurement at age 13 instead. Changes in social exclusion between waves therefore reflect changes between age 13 and 20. Social exclusion was reliable across waves (Cronbach’s α _*age13*_ = 0.87, Cronbach’s α _*age20*_ = 0.88).

*Employment* and *University Attendance* are two “turning points” measured at age 20 that are likely to elicit changes in adult status and responsibility (Ford and Schroeder 2010; Uggen 2000). Respondents were asked whether or not they had found new employment and/or started university in the 2 years prior to the survey.

*Deviant Behavior* measured whether or not the respondent engaged in 16 antisocial behaviors in the year prior to the survey at ages 17 and 20. This includes fare dodging, drug dealing, vandalism, theft, and violence. We created a variety score that reflects the number of different types of deviant acts the respondent engaged in. Variety scores are considered a more valid measure of the intensity of deviance compared to frequency measures of the total number of deviant acts (Sweeten 2012).

*Police Legitimacy* was measured using three items that capture aspects of procedural justice, fair decision-making, and confidence in police effectiveness (Sunshine and Tyler 2003). Items included were as follows: “Police treat people with dignity and respect,” “I’m confident that the police can do their job well,” and “police apply the rules consistently to different people.” Responses were measured on a four-point Likert-type scale ranging from “fully untrue” to “fully true.” The police legitimacy scale was reliable across waves (Cronbach’s α _*age17*_ = 0.87, Cronbach’s α _*age20*_ = 0.84).

*Perceived Peer Disapproval of Violence* was measured at ages 17 and 20 using two situational vignettes, in which respondents answer a series of standardized questions relevant to aggressive decision-making. The vignettes portray two situations of reactive (non-extremist) physical aggression and verbal aggression respectively. Four items are relevant for capturing peer attitudes towards violence (e.g. “Would your best friends admire you and think you were cool because of this?” “Would your best friends think it is bad to do this?”). The vignettes and items were adapted from instruments used in the KFN studies (Wetzels et al. 2001) and Denver Youth Survey (Huizinga, 1988–1992). Responses from both vignettes were combined into a single scale, where higher values indicated greater peer disapproval of violence (Cronbach’s α = 0.89 for both waves).

*Competent Conflict Coping Skills* reflect the extent to which individuals are able to cope with conflict and adverse experiences (Agnew 2006). Competent conflict coping skills are measured at age 17 using four items, with agreement on a five-point Likert-type scale ranging from “never” to “very often”. Items include “I listen carefully so that there are no misunderstandings” and “I try to control my anger.” The scale is reliable, with a Cronbach’s α of 0.77 at age 17 and 0.69 at age 20.

*Low Self-Control* was measured using a 10-item scale adapted from Grasmick et al. (1993). The scale consists of five subdimensions of two items each: impulsivity, self-centeredness, risk-seeking, preference for physical activities, and short temper. Reliability was acceptable across waves (Cronbach’s α _*age17*_ = 0.73, Cronbach’s α _*age20*_ = 0.74).

#### Sociodemographic Control Variables

Three sociodemographic variables were included: gender, religious denomination, and migrant background. *Gender* was coded 0 for females and 1 for males. We created a dichotomous variable measuring whether the respondent identified as Muslim at age 15. All other religious or non-religious backgrounds (i.e., Christian [Protestant, Catholic, Orthodox], Jewish, Buddhist, Hindu, none) were coded as 0. Finally, *migrant background* was coded 0 for youths with at least one parent born in Switzerland, and 1 for those with both parents born outside of Switzerland.

### Analytical Procedure

The analysis proceeds in two stages. In the first stage, we describe significant changes in an individual’s extremist attitudes. One common approach to evaluating change over time is to assess changes in variables between time points using t-tests or ANOVAs (Zahra et al. 2016). However, these statistics only speak to average *group* change, and cannot tell us to what extent *individuals* experienced meaningful and statistically significant change (Guhn et al. 2014). Therefore we used the Reliable Change Index [RCI] to measure and evaluate change in violent extremist attitudes between ages 17 and 20 (Jacobson and Truax 1991). The RCI is a psychometric concept that is used to evaluate to what extent change in a construct is “reliable” (i.e. statistically significant), accounting for change due to random fluctuation or measurement error (Jacobson et al. 1991). Here we use the Jacobson and Truax (1991) RCI (1), which is calculated by dividing the difference between age 20 and age 17 scores (*x*_*2*_-*x*_*1*_) by the standard error of measurement difference (*S*_*diff*_) (Guhn et al. 2014).

1 $$RCI = ~\frac{{x_{2} - ~x_{1} }}{{S_{{diff}} }}$$

The standard error of measurement of difference reflects the expected distribution of change scores if no change were to occur (Jacobson and Truax 1991). The standard error of difference is calculated using the following formula (2):

2 $$S_{{diff}} = ~\sqrt {2\left( {s_{{1\sqrt {1 - r_{{xx}} } }} } \right)^{2} }$$

where by *s*_*1*_ represents the standard deviation of the score at age 17, and *r*_*xx*_ represents the test–retest reliability, or correlation coefficient, between scores at age 17 and 20.

In essence, the RCI is an expression of the real differences in scores, accounting for variability and reliability in the measure (Zahra et al. 2016). The RCI is equivalent to an analysis based on standardized z-scores, where values greater or less than |1.96| indicate significant differences in scores between time points. Although typically used to assess changes in clinical samples during interventions, the RCI can be a useful tool for identifying individuals who have experienced meaningful (“clinical”) changes in violent extremist attitudes. In other words, the RCI can identify individuals whose extremist attitudes have declined from high (“extremist”) to low (“non-extremist”) levels between ages 17 and 20, or vice versa.

In the second stage, we aim to explain between- and within-individual variation in mean scores of violent extremist attitudes from age 17 to 20. In order to do so, we use hybrid effects regressions that can differentiate and estimate both between- and within-individual effects of social and psychological factors on violent extremist attitudes (Allison 2009; Curran and Bauer 2011; Schröder 2018; Schunck 2013). The formula (3) for the hybrid model is presented below:

3 $$y_{{it}} = b_{0} + b_{1} \left( {x_{{it}} - \bar{x}_{i} } \right) + b_{2} \bar{x}_{i} + b_{3} c_{i} + \mu _{i} + \varepsilon _{{it}}$$

The hybrid model estimates support for violent extremism (*y*) for individual *i* at time *t* (age). The term *b*_0_ represents the intercept, which reflects the average level of violent extremist attitudes when all predictors are equal to 0. The term *b*_*1*_ represents the within-individual (fixed) effect, *b*_*2*_ represents the between-individual effect, and *b*_*3*_ represents the effect for time-invariant variable *c* for individual *i* (e.g. gender). The term *x*_it_ represents the value for independent variable *x* for individual *i* at time *t*, and *x̄*_*i*_ measures the average value of variable *x* for individual *i*. The between-individual effect is estimated using this mean value (*x̄*_*i*_). In order to estimate within-individual effects, the independent variable is centered around the mean for that subject (*x*_it_−*x̄*_*i*_). The resulting value is known as the deviation score, and it reflects the variability of the variable at time *t*, relative to the average value for individual *i* (see Schröder 2018). The terms *μ*_*i*_ and ε_*it*_ represent error terms for individual *i* and individual *i* at time *t*, respectively.

The hybrid model will be able to decompose to what extent social or psychological risk factors can explain differences in violent extremist attitudes between individuals as well as mean changes over time within individuals. For example, the hybrid model can estimate whether an individual’s support for violent extremism is lower among those who report overall higher levels of police legitimacy (between-individual effects), and whether an individual’s support is lower than usual at a given age when he/sche reports higher police legitimacy for that year (within-individual effects). Since hybrid models require the calculation of cluster means and deviation scores (Schunk, 2013), it is necessary to restrict the sample to observations for which all information is available (n = 910).

It is important to note that the RCI and hybrid regressions capture and assess different metrics of “change” in violent extremist attitudes. The RCI measures “significant” or “clinical” change based on a pre-established statistical threshold and accounting for measurement error (Zahra et al. 2016). As a result, the picture of change over time is likely to be more conservative compared to the overall variation in mean scores captured by the hybrid models.

An analysis of the participants excluded due to listwise deletion showed no significant differences at age 17 and 20 in levels of violent extremist attitudes, deviant behavior, low self-control, police legitimacy, peer disapproval of violence, social exclusion, and experiences of relational loss or trauma. At age 17 (but not age 20), excluded respondents were more likely to have experienced achievement loss and criminal justice contact. Excluded respondents were significantly less likely to be employed, have competent conflict coping skills (at age 20), more likely to have a migrant background, and identify as Muslim.^2^

The data and statistical code used in the current study are available from the corresponding author on reasonable request.

## Results

### Descriptive Results

**Fig. 1:**
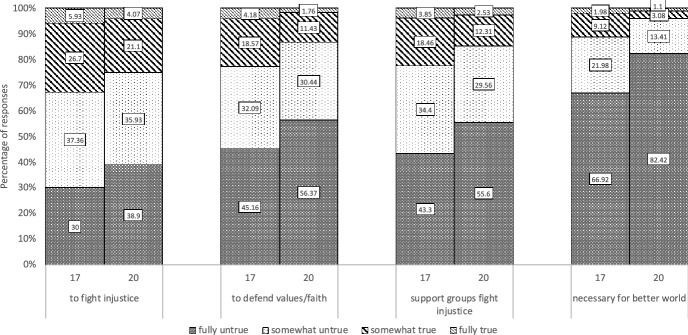
Percentage of responses by response category to statements on the use of violence to achieve collective goals (n = 910)

Tables 2 and 3 displays the descriptive statistics and bivariate correlations for all variables at ages 17 and 20. In accordance with the age-crime curve, Table 2 shows that the average levels of deviant behavior declined between ages 17 (*M* = 1.93) and 20 (*M* = 1.50). The average level of violent extremist attitudes also declined over time (*M*_*age17*_ = 1.80, *M*_*age20*_ = 1.58). When comparing mean scores between ages 17 and 20, 50.99 percent reported a decline in violent extremist attitudes to some degree. Among those reporting a decline, the percentage change ranged from − 6.25 percent to − 73.33 percent.^3^ 22.42 percent of respondents reported no (zero percent) change in attitudes between ages 17 and 20. The remaining 26.59 percent reported an increase in support for violent extremism, ranging from 8.33 to 225 percent. To describe these overall differences further, we examined the changes in response categories for each item of the violent extremist attitudes scale between ages 17 and 20. The percentages for each response category per item and age are displayed in Fig. 1. The results show that overall, the majority of respondents (66% or above) rated the statements as “somewhat untrue” or “fully untrue”, with this proportion of respondents increasing over time. This suggests that in general support for violent extremism remains low between ages 17 and 20.

**Table 2:** Descriptive statistics for variables used in the analyses

	t1: age 17		t2: age 20			
Variables	M/%	SD	M/%	SD	Min	Max
Gender (1 = male)	49%	0.50			0	1
Migrant background	45%	0.50			0	1
Religious denomination (1 = Muslim)	16%	0.37			0	1
Employed			56%	0.50	0	1
Attending university			26%	0.44	0	1
Achievement loss	28%	0.45	26%	0.44	0	1
Relational loss	38%	0.49	56%	0.50	0	1
Victimization	9%	0.28	11%	0.32	0	1
Contact with CJS	7%	0.26	11%	0.31	0	1
Violent extremist attitudes	1.80	0.67	1.58	0.59	1	4
Low self-control	2.21	0.42	2.06	0.42	1	4
Coping skills	3.58	0.76	3.74	0.77	1	5
Police legitimacy	2.60	0.75	2.58	0.70	1	4
Perceived peer disapproval of violence	1.26	0.64	1.40	0.64	− 0.25	2.75
Social exclusion^a^	1.50	0.53	1.50	0.58	1	4
Deviant behavior	1.93	1.88	1.50	1.57	0	16

*SD* standard deviation.

N = 910

^a^Social exclusion was measured at age 13 instead of age 17

**Table 3:** Bivariate correlations for all variables included in the analyses

		1	2	3	4	5	6	7	8	9	10	11	12	13
1	Violent extremist attitudes (age 17)	1.00												
2	Violent extremist attitudes (age 20)	.42***	1.00											
3	Deviant behavior (age 17)	.24***	.26***	1.00										
4	Deviant behavior (age 20)	.16***	.29***	.50***	1.00									
5	Low self-control (age 17)	.33***	.24***	.40***	.25***	1.00								
6	Low self-control (age 20)	.23***	.29***	.28***	.29***	.59***	1.00							
7	Police legitimacy (age 17)	− .25***	− .21***	− .33***	− .27***	− .32***	− .26***	1.00						
8	Police legitimacy (age 20)	− .22***	− .21***	− .28***	− .30***	− .27***	− .26***	.52***	1.00					
9	Perceived peer disapproval of violence (age 17)	− .37***	− .25***	− .33***	− .24***	− .40***	− .30***	.31***	.24***	1.00				
10	Perceived peer disapproval of violence (age 20)	− .28***	− .31***	− .25***	− .25***	− .27***	− .34***	.22***	.21***	.64***	1.00			
11	Coping skills (age 17)	− .24***	− .13***	− .17***	− .10**	− .35***	− .25***	.24***	.17***	.30***	.20***	1.00		
12	Coping skills (age 20)	− .13***	− .12***	− .06	− .04	− .20***	− .18***	.09**	.14***	.12***	.10**	.46***	1.00	
13	Social exclusion (age 13)	.03	.03	.03	.06	.05	.05	− .04	− .06	.02	.05	− .08*	− .06	1.00
14	Social exclusion (age 20)	.13***	.21***	.07*	.19***	.00	.13***	− .08*	− .12***	.02	.02	− .05	− .07*	.31***
15	Employed (age 20)	− .08*	− .01	.01	.06	.08*	.05	− .09**	− .08*	− .04	− .05	− .03	.04	.02
16	Attending university (age 20)	− .10**	− .01	− .01	− .01	− .15***	− .21***	.11***	.11***	.18***	.19***	.11**	.11***	− .04
17	Achievement loss (age 17)	.12***	.12***	.20***	.12***	.13***	.14***	− .12***	− .09**	− .19***	− .19***	− .14***	− .04	.08*
18	Achievement loss (age 20)	.11***	.12***	.16***	.22***	.15***	.13***	− .13***	− .15***	− .15***	− .13***	− .13***	− .09**	.03
19	Relational loss (age 17)	− .02	.00	.16***	.09**	.16***	.11**	− .10**	− .10**	− .05	− .06	− .02	.01	.10**
20	Relational loss (age 20)	.02	.04	.11**	.06	.13***	.10**	− .13***	− .10**	− .08*	− .10**	− .05	.04	.08*
21	Victimization (age 17)	.04	.02	.16***	.07*	.10**	.03	− .14***	− .08*	− .04	− .02	− .06	− .07*	.07*
22	Victimization (age 20)	.02	.04	.16***	.19***	.09**	.13***	− .09**	− .11**	− .01	− .07*	.01	− .01	.05
23	Contact with CJS (age 17)	.08*	.07*	.31***	.19***	.15***	.07*	− .21***	− .10**	− .19***	− .13***	− .11***	− .06	.04
24	Contact with CJS (age 20)	.12***	.10**	.27***	.30***	.20***	.14***	− .18***	− .21***	− .24***	− .20***	− .11**	− .04	− .04
25	Gender (1 = male)	.28***	.28***	.25***	.22***	.12***	.14***	− .15***	− .09*	− .47***	− .42***	− .05	.01	− .07*
26	Religious denomination (1 = Muslim)	.11***	.05	.00	− .03	.09**	.07*	− .03	− .06	− .13***	− .12***	− .06	− .03	− .03
27	Migrant background	.10**	.02	− .06	− .12***	.07*	.04	− .03	− .01	− .11***	− .10**	− .07*	− .07*	.06

		14	15	16	17	18	19	20	21	22	23	24	25	26	27
14	Social exclusion (age 20)	1.00													
15	Employed (age 20)	− .02	1.00												
16	Attending university (age 20)	− .03	− .26***	1.00											
17	Achievement loss (age 17)	.13***	− .04	− .19***	1.00										
18	Achievement loss (age 20)	.11**	− .02	− .11***	.32***	1.00									
19	Relational loss (age 17)	.04	.12***	− .05	.08*	.01	1.00								
20	Relational loss (age 20)	.05	.06	− .02	.05	.02	.19***	1.00							
21	Victimization (age 17)	.05	.05	.02	.14***	.13***	.12***	.07*	1.00						
22	Victimization (age 20)	.07*	.06	− .07*	.08*	.07*	.14***	.18***	.12***	1.00					
23	Contact with CJS (age 17)	.05	.02	− .09**	.12***	.12***	.05	.02	.11***	.13***	1.00				
24	Contact with CJS (age 20)	− .01	.04	− .09**	.10**	.14***	.03	.08*	.10**	.14***	.30***	1.00			
25	Gender (1 = male)	− .02	− .10**	− .05	.08*	.10**	− .06	− .06	.02	− .04	.14***	.20***	1.00		
26	Religious denomination (1 = Muslim)	− .03	.01	− .16***	.12***	.10**	− .06	− .07*	− .02	− .02	.06	.03	− .03	1.00	
27	Migrant background	.03	.04	− .14***	.20***	.06	− .04	.01	.05	− .04	− .02	.02	− .01	.36***	1

*p < 0.05, **p < 0.01, ***p < 0.001

Table 3 shows that violent extremist attitudes at age 17 were moderately correlated with violent extremist attitudes at age 20 (r = 0.42, *p* < 0.001). Notably, this is relatively less stable over time compared to e.g., low self-control (r = 0.59, *p* < 0.001). The strongest strain-related correlates with violent extremist attitudes at age 20 were achievement loss (r_age20_ = 0.12, p < 0.001) and contact with the criminal justice system (r_age20_ = 0.10, *p* < 0.01). More proximal individual correlates showed relatively larger concurrent associations with violent extremist attitudes at ages 17 and 20. The strongest correlate of violent extremist attitudes at age 20 was peer disapproval of violence (r_age20_ = -0.31, *p* < 0.001).

### Changes in Violent Extremist Attitudes

**Fig. 2:**
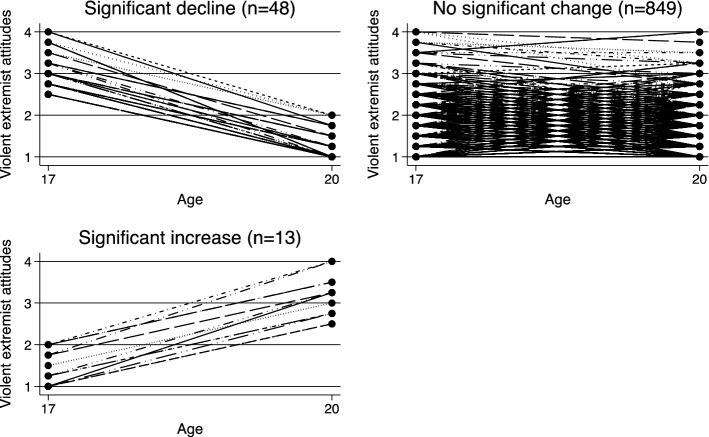
Reliable Change Index for violent extremist attitudes between ages 17 and 20 Note. An RCI score of < − 1.96 indicates a significant decline, an RCI score of > 1.96 indicates a significant increase, and an RCI score between ± 1.96 indicates no significant change between time points

While Table 2 shows that there are differences in the average level of violent extremist attitudes between ages 17 and 20, this does not provide information about the extent to which individuals experienced meaningful and significant change over time. In order to do so, we evaluated to what extent individual changes were clinically significant using the Reliable Change Index. The results are displayed in Fig. 2. In line with the descriptive results, the RCI scores indicated that for the vast majority of individuals violent extremist attitudes did not experience clinically significantly change between ages 17 and 20 (93.2%, n = 849). Within this stable group, overall levels of support for violent extremism were consistently low. Among those in the stable group, 75% of youths at age 17 and 83% at age 20 scored an average of 2 or below (low support) on the violent extremist attitudes scale. By contrast, 1.3% and 0.59% at ages 17 and 20, respectively, within the stable group scored an average above 3 (strong support) on the violent extremist attitudes scale. A small number of respondents reported a significant decline in violent extremist attitudes (5.3%, n = 48), and only 13 respondents (1.4%) reported clinically significant increases during this time.

Table 4 reports the descriptive characteristics for each RCI category by age. This provides some insight into the factors that may be associated with meaningful and significant declines or increases in violent extremist attitudes. Significant differences in characteristics are estimated using paired sample t-tests. The results for the declining group suggest that participants who experienced significant declines also reported significantly lower levels of deviant behavior, higher levels of self-control, and higher levels of peer disapproval of violence at age 20. Those in the declining group also experienced more relational loss at age 20 compared to age 17 (*M*_*age17*_ = 31% vs. *M*_*age20*_ = 58%, t = -2.91, *p* < 0.001). Those in the stable group reported significantly lower levels of deviant behavior and low self-control, and higher peer disapproval of violence, competent coping skills, relational loss, and victimization at age 20. We found no significant differences in characteristics for the increasing group, which may be due to the low number of respondents in this group (n = 13).

**Table 4:** Descriptive characteristics of declining, stable, and increasing violent extremist attitude groups

	RCI declining (n = 48)			RCI stable (n = 849)			RCI increasing (n = 13)		
Variables	Age 17	Age 20	t-value	Age 17	Age 20	t-value	Age 17	Age 20	t-value
Violent extremist attitudes	2.95	1.24		1.74	1.58		1.44	3.15	
Deviant behavior	1.81	1.13	2.60*	1.94	1.51	7.30***	1.69	2.46	1.55
Low self-control	2.36	2.04	5.72***	2.20	2.06	10.67***	2.13	2.13	< 0.01
Police legitimacy	2.41	2.35	0.47	2.61	2.60	0.26	2.49	2.15	1.73
Perceived peer disapproval of violence	1.05	1.37	− 3.72***	1.27	1.41	− 7.47***	1.24	1.08	1.19
Coping skills	3.31	3.47	− 1.43	3.60	3.76	− 5.91***	3.54	3.44	0.46
Social exclusion	1.42	1.39	0.40	1.50	1.51	− 0.44	1.31	1.32	− 0.08
Achievement loss	33%	33%	< 0.01	28%	26%	1.12	38%	54%	− 0.81
Relational loss	31%	58%	− 2.91**	39%	56%	− 8.12***	38%	54%	− 1.00
Victimization	10%	10%	< 0.01	8%	12%	− 2.43*	15%	0%	1.48
Contact with CJS	8%	15%	− 1.14	7%	10%	− 2.78**	15%	15%	< 0.01
Employed		35%			57%			62%	
Attending university		23%			26%			23%	
Gender (1 = male)		52%			48%			62%	
Migrant background		56%			44%			15%	
Religious denomination (1 = Muslim)		17%			16%			15%	

RCI = Reliable Change Index. Paired sample t-tests were conducted to evaluate differences between ages within each category. Mean values or proportions (%) are presented.

*p < .05, **p < .01, ***p < .001

### Explaining Violent Extremist Attitudes

Table 5 presents the results for the hybrid model that decomposes the between- and within-individual effects of distal and proximal factors on violent extremist attitudes. The results show that out of the different forms of strain or significance loss, only social exclusion (b = 0.15, *p* < 0.001) was associated with higher levels of violent extremist attitudes between individuals. Regarding individual attitudes and propensities, the results show that overall, those who support violent extremism are also more likely to report lower self− control (*b*_*between*_ = 0.25, *p* < 0.001), less competent coping skills (*b*_*between*_ = − 0.06, *p* < 0.05), lower police legitimacy (*b*_*between*_ = − 0.10, *p* < 0.001), lower peer disapproval of violence (*b*_*between*_ = − 0.15, *p* < 0.001), and more deviant behavior (*b*_*between*_ = 0.04, *p* < 0.01). Individuals who are employed at age 20 are less likely to support violent extremism (*b*_*between*_ = − 0.06, *p* < 0.05).

**Table 5:** Hybrid regression models examining between- and within-individual effects on violent extremist attitudes (ages 17 and 20)

	Between-individual effects		Within-individual effects	
Variables	b	SE	b	SE
Intercept	1.57***	[0.19]		
Gender (1 = male)	0.20***	[0.04]		
Religious denomination (1 = Muslim)	0.08	[0.04]		
Migrant background (1 = both parents born abroad)	0.02	[0.03]		
Employment (age 20)	− 0.06*	[0.03]		
University (age 20)	0.05	[0.04]		
Achievement loss	0.02	[0.05]	0.01	[0.04]
Relational loss	− 0.07	[0.04]	< 0.01	[0.04]
Victimization	− 0.03	[0.07]	< 0.01	[0.06]
Contact with CJS	− 0.11	[0.07]	− 0.05	[0.07]
Social exclusion	0.15***	[0.03]	0.04	[0.03]
Low self-control	0.25***	[0.05]	0.20**	[0.06]
Coping skills	− 0.06*	[0.02]	− 0.06*	[0.03]
Police legitimacy	− 0.10***	[0.03]	< 0.01	[0.03]
Perceived peer disapproval of violence	− 0.15***	[0.03]	− 0.15***	[0.04]
Deviant behavior	0.04**	[0.01]	0.02	[0.01]
Wave (1 = age 20)			− 0.14***	[0.03]
σ_u_	0.30			
σ_e_	0.47			
ICC (ρ)	0.29			
R^2^ overall	0.25			
R^2^ between	0.30			
R^2^ within	0.14			

Unstandardized coefficients are reported, with standard errors in brackets.

*ICC*  interclass correlation. *SE*  standard error. Hybrid models estimate within-individual effects using random effects models.

*p < .05; **p < .01; ***p < .001. N (observations) = 1820. N (individuals) = 910

The results for within-individual effects show that during periods when individuals report lower self-control (*b*_*within*_ = 0.20, *p* < 0.01), lower competent coping skills (*b*_*within*_ = − 0.06, *p* < 0.05), and lower peer disapproval (*b*_*within*_ = − 0.15, *p* < 0.001), they are more likely to support violent extremism. By contrast, violent extremist attitudes are not significantly higher during periods of strain or significance loss. The results also confirm that on average violent extremist attitudes were lower at age 20 compared to age 17 (b = − 0.14, *p* < 0.001). Taken together, adverse life events and individual characteristics account for 30% of the between-individual variance. The proportion of within-individual variance explained is relatively lower at 14%.^4^

## Discussion

In this article, we aimed to identify and explain patterns of change in violent extremist attitudes during the transition to adulthood (between ages 17 and 20). We formulate three main conclusions that can be drawn from our findings and discuss their implications for research and policy related to violent extremist attitudes and radicalization more generally.

First, in line with our expectation, we found that average levels of support for violent extremism were lower at age 20 compared to age 17. When examining individual change according to the Reliable Change Index, only a small percentage (1.4%) of respondents reported a clinically significant increase in support across waves. Instead, the vast majority of respondents reported stable (93.2%) or significantly declining (5.3%) support for violent extremism over time. To recall, the RCI presents a different metric of “change” over time compared to average levels of change or the incremental mean change assessed in the hybrid models. Change in mean scores assessed in the hybrid models can be either large or small. The RCI incorporates a threshold to define “significant” or “meaningful” differences in support for violent extremism while accounting for measurement error, and as a result lead to a more conservative picture of change over time. The vast majority of respondents reported stable or non-significant change between ages 17 and 20 according to the RCI, but actually 77.58% of the respondents reported some amount of change between the two waves. What the RCI shows is that most of these changes in violent extremist attitudes are usually small and more incremental, and that more dramatic “significant” changes are uncommon.

Overall, this suggests that extremist attitudes follow a trajectory similar to the aggregate age-crime curve, whereby support increases during adolescence and decreases as individuals transition into adult roles and responsibilities. This finding is in line with previous cross-sectional evidence that shows a negative relationship between age and violent extremist attitudes and behaviors (Wolfowicz et al. 2019). Research on political behavior has demonstrated a similar inverse U-shaped curve for “unconventional” attitudes and actions, such as support for illegal demonstrations or use of political violence (Watts, 1999). However, theoretical models of radicalization are generally “ageless” in that they rarely specify age-graded patterns or effects across the life course (e.g. Agnew 2010; Bouhana and Wikström 2010; Kruglanski et al. 2019; Moghaddam 2005). Our results provide further support for the development and integration of life course and developmental perspectives into research on violent extremist attitudes and radicalization (see e.g., Simi et al. 2016). For example, if trajectories of violent extremist attitudes follow the age-crime curve, the risk of radicalization may be the highest during adolescence.

Second, only some measures of personal strain and significance loss were associated with between-individual differences in violent extremist attitudes. Our study investigated a wide range of personal strains, however only achievement loss (i.e. academic failure, failure to find training, apprenticeship), contact with the criminal justice system, and feelings of social exclusion were associated with overall higher levels of support for violent extremism in bivariate models. The former is not surprising given the importance of completing education and finding employment during this transitional stage of the life course. Regarding social exclusion, our findings are also in line with previous research that shows a between-individual association between low social integration (i.e. ostracism, marginalization, discrimination, exclusion) and violent extremist attitudes (Lyons-Padilla et al. 2015; Pauwels and De Waele 2014; Pfundmair 2018; Pretus et al. 2018; Wolfowicz et al. 2019).

The differences in effects between types of negative life experiences suggest that the relationship between strains or significance loss may be age-graded, whereby certain negative experiences can be more “disruptive” and generate negative emotions and uncertainty at different stages of the life course. For example, while achievement loss may be more disruptive during the transition to adulthood, relational loss or divorce may be a more salient risk factor in later adulthood when family relationships play a more central role in the formation of adult identity and status (Benson and Furstenberg 2007). Future research should examine additional life events and experiences that are most salient to young adults during the transition to adulthood. In addition, previous research on GST suggests that the effects of strain on criminal or extremist outcomes are also conditional on individual attitudes and propensities (Mazerolle and Maahs 2000; Nivette et al. 2017). Additional longitudinal research is needed to evaluate the direct and conditional effects of negative life events on violent extremist attitudes during different stages of adolescence and adulthood.

We did not find robust evidence that support for violent extremism was higher during periods of greater strain (within-individual effects). These findings suggest that individuals who support violent extremism are also likely to experience certain strains, but that increases in these strains do not necessarily lead to increases in support. Alternatively, within-individual effects may play out in the short- or longer-term, as emotional reactions and coping are likely to occur immediately after the event (see e.g., Pretus et al. 2018), or psychological processes of loss and trauma may be delayed or progress slowly over time (Updegraff and Taylor 2000). A 3-year window may not be suitable for capturing either short- or longer-term processes.

Third, in line with our expectations, we found that between- as well as within-individual differences in support for violent extremism were related to several social and individual characteristics. Low self-control, poor coping skills, low perceived police legitimacy, high peer approval of violence, and own deviant behavior were all associated with relatively more violent extremist attitudes. Irrespective of these between-individual differences, increases in self-control, competent coping skills, and prosocial peers were associated with decreases in support for violent extremism. Additionally, individuals who were employed at age 20 reported lower levels of support. These changes all reflect aspects of psychosocial maturity and the formation of prosocial bonds and responsibilities in adulthood that are seen as key to desistance from crime (Mulvey et al. 2004; Rocque 2015; Sampson and Laub 1993). This means that our results support the notion that knowledge and evidence on desistance from crime are relevant for understanding deradicalization processes (Decker and Pyrooz 2015; Della Porta and LaFree 2012; Freilich and LaFree 2015). Existing research on effective interventions for criminal desistance (e.g., Koehler et al. 2013) and disengagement from gangs (e.g., Roman et al. 2017) may therefore be fruitful avenues for developing programs aimed at reducing support for violent extremism and fostering deradicalization. More specifically, our results suggest that interventions targeting the development of self-control and coping skills, such as perspective-taking and empathy, can reduce support for violent extremism (see e.g. Feddes et al. 2015).

It is important to note that while we find parallels between criminological theories and support for violent extremism, the current study does not explicitly investigate whether or not the same processes could also account for changes in offending. It may be that certain factors, such as types of strain or victimization, psychosocial characteristics, or peer contexts, uniquely motivate support for criminal violence or other offending behaviors, but not extremist violence. Research on violent extremism would therefore benefit from more rigorous comparisons of risk factors for extremist and criminal offenders (see e.g. Horgan et al. 2016; Liem et al. 2018; Parkin and Freilich 2015).

Taken together, our results demonstrated the importance of distinguishing between- and within-individual effects on support for violent extremism. The vast majority of empirical research on violent extremist attitudes examines between-individual variation, with the assumption that these effects can also be applied to within-individual changes. The current study demonstrated that this is not always a fair assumption. While low self-control, coping skills, and peer disapproval of violence were associated with both between- and within-individual differences in support for violent extremism, social exclusion, police legitimacy and deviant behavior were associated with only between-individual differences. The null findings for social exclusion may be an artefact of measurement, as we used social exclusion measured at age 13 as a proxy for age 17 attitudes. We therefore hesitate to draw conclusions about within-individual effects of social exclusion on violent extremist attitudes. Researchers should assess how different forms of social exclusion and feelings of discrimination influence changes in support for violent extremism in the short- and long-term. The null within-individual effects for police legitimacy are in line with previous research on self-reported offending over time (Kaiser and Reisig 2019). This implies that while low police legitimacy may serve as an important risk factor for identifying youths at risk for radicalization, short-term fluctuations in police legitimacy are insufficient for generating changes in support for violent extremism. Furthermore, the proportion of between-individual variance explained was twice that of the proportion of within-individual variance explained (Table 5: R^2^ between = 0.30, R^2^ within = 0.14).

The results from the Reliable Change Index suggest that individuals who reported significant increases were not remarkably different from their peers at age 17 in terms of psychosocial characteristics or deviant behavior, nor did we detect significant changes in risk factors between ages 17 and 20 (see Table 4). While this may be in part due to the small group size, it suggests that identifying the risk of radicalization and the factors that motivate significant change can be a serious challenge in both research and practice. This means that there are other within-individual mechanisms that are not accounted for in the current model, or that factors that influence incremental changes, as shown in the hybrid models, differ from the factors that contribute to the significant, “clinical” changes measured by the RCI. Future research is needed to shed more light on these.

### Limitations and Conclusions

The current study is one of the few studies with prospective longitudinal measures of support for violent extremism among a diverse sample of young adults. It enabled the analysis of between- as well as within-individual differences in violent extremist attitudes. However, besides these strengths there are important limitations to consider.

First, while we were able to evaluate within-individual variation over time, we were limited to only two waves. Ideally, additional time points over a longer age period in the lives of adolescents and early adults are needed. These would provide more information for estimating deviations from the individual mean and analyzing changes over the life-course. A small number of respondents reported stable but “high” levels of support for violent extremism between ages 17 and 20 (between 0.59 and 1.3%). A longer time frame before adolescence and into adulthood can better determine whether attitudes overall follow the traditional inverse-U shape of the age-crime curve, and to what extent different extremist “careers” exist similar to criminal behavior (e.g. early onset, late starters, stable chronics, categories distinguished for criminal careers, see DeLisi and Piquero 2011).

This is also important because previous research suggests that the relationship between age and violent extremism might vary by ideological orientation. For example, using the American Terrorism Database, Smith (1994) found that right-wing terrorists tended to be older than left-wing terrorists, whereas Chermak and Gruenewald (2015) found that suspects associated with Al Qaeda and affiliated movements were significantly older than right- and left-wing suspects in the United States. These age differences may signify that there are important differences between ideologies with regard to the onset and trajectory of extremism over the life course. Future research should aim to examine both general and ideologically-specific attitudes towards the use of extremist violence in order to determine whether and to what extent the radicalization process varies across ideologies and groups.

Second, attrition between ages 17 and 20 meant that we could only include respondents who participated in both waves. An analysis of attrition between participants and dropouts at age 20 suggested that dropouts differ significantly on certain characteristics (i.e. migrant background, not employed, higher prevalence of achievement loss). The results may therefore be biased towards non-migrant, employed respondents. Although the current study does have an ethnically and religiously diverse community sample, longitudinal research on radicalization is needed in more and different populations. Further, dropouts between ages 17 and 20 reported significantly higher support for violent extremism compared to those that participated in both waves. This may indicate that the results are biased towards respondents with lower initial levels of support for violent extremism. Nevertheless, imputed results were substantively similar (see Online Appendix Table A1), which suggest that the factors related to changes in support for violence are not affected by this selective attrition. To get a better understanding of high-risk samples, future research might include offender or prison populations or focus specifically on youth considered at risk for right-, left-, or religiously-motivated extremism.

A third limitation is that we were not able to measure all mechanisms that link significance loss and strain to violent extremist attitudes (e.g. perceived significance loss, sense of uncertainty, identity-seeking). While our goal in this paper was not to explicitly test these theories, the omission of conditional effects and mediation processes may not adequately capture the indirect processes by which strain influences violent extremist attitudes. Relatedly, the current study was not able to capture a range of potential mechanisms that are theoretically relevant for understanding the process of (de)radicalization, such as structural or neighborhood dynamics or more extensive characteristics of peer networks and influences (see Lösel et al. 2018; Wolfowicz et al. 2019). For example, we are not able to incorporate the respondent’s exposure to extremist peer groups and messaging, both offline and online (Frissen 2021; Pauwels and Schils 2016). Our measure of peer influence captures self-reported perceptions of peer disapproval of *general* violence, which may to some extent reflect a projection of their own attitudes and behaviors (Walters 2019). Future research should aim to measure the social networks of respondents, including both self- and peer-reported attitudes towards general and extremist violence.

Overall, our results show that criminological theory and knowledge on criminal desistance are useful for understanding changes in violent extremist attitudes during the transition to adulthood. For young people in Zürich, this stage of the life course was characterized by increases in psychosocial maturity, more prosocial peers, and less deviant behavior, which in turn was associated with lower support for violent extremism. While our research points towards a general declining trend into early adulthood, more research is needed to identify and understand differential trajectories of violent extremist attitudes over the life course.

## Supplementary Information

Below is the link to the electronic supplementary material.

Supplementary file1 (DOCX 27 kb)

## Footnotes

1 63 youths participated at age 20, but not age 17.

2 For comparison, we also estimated the hybrid model using multiple imputation for missing data. In this case, 20 imputed datasets and estimated coefficients and standard errors using Rubin’s (1987) rules. The results remained substantively the same, with a few minor differences. In the hybrid model with imputed values, the between-individual coefficient for relational loss is significant (b = -0.08, p < .05) and the within-individual coefficient for deviant behavior is significant (b = 0.04, p < .01). Full imputed results are available in the online appendix (see Table A.1).

3 Percentage change was calculated using the following formula: ((x_age20_-x_age17_)/x_age17_)*100.

4 As an additional analysis, we estimated the between- and within-individual effects of strain variables as variety scores instead of dichotomized prevalence scores, and using a combined strain variety scale. A higher score on each type of strain, or on the combined scale, indicates exposure to different types of strain. The results remained substantively similar, with the exception that individuals who reported more relational loss were overall less likely to support violent extremism (between-individual effects). The results for between-individual overall strain are also negatively related to violent extremist attitudes, which is likely driven by relational loss effects. Although this is counter-intuitive, this may be explained by the notion that more socially connected adolescents have a broader social network, and therefore may be more likely to experience break ups with friends and significant others more generally. In addition, these break ups may be more common during the transition to adulthood as youths are spending more time in new social settings at work or university. The results are presented in the Online Appendix (Tables A.2 and A.3).
