# Supplementary material for: Understanding Changes in Violent Extremist Attitudes During the Transition to Early Adulthood
Source: J Quant Criminol. 2021 Jul 8;38(4):949–78. doi: 10.1007/s10940-021-09522-9 (PMC9626430; doi:10.1007/s10940-021-09522-9)
Supplement: Supplementary file 1 — Supplementary file1 (DOCX 27 kb) [file 10940_2021_9522_MOESM1_ESM.docx]

**Online Appendix - Understanding changes in violent extremist attitudes during the transition to early adulthood**

*1. Results using multiple imputation*

Inspection of missing data patterns reveals that missingness is conditional on observed data included in the analyses (e.g. low self-control, achievement loss, migrant background), and can therefore be classified as missing at random [MAR]. Arguably, non-response will not bias the estimates if these variables are included to control for the probability of missingness (Gelman & Hill, 2006). Nevertheless, we re-estimated all models with imputed values as a robustness check. Imputation was conducted using multiple imputation with chained equations, which can account for different types of data, including categorical, bounded, and continuous variables (Azur et al., 2011). We imputed 20 complete datasets using all variables included in the analyses, and re-estimated the coefficients and standard errors using Rubin’s (1987) rules.

Table A.1. Hybrid regression models using multiple imputation (ages 17 and 20)

|  | Between-individual effects | | Within-individual effects | |
| --- | --- | --- | --- | --- |
| Variables | b | SE | b | SE |
| Intercept | 1.44*** | [0.17] |  |  |
| Gender (1=male) | 0.18*** | [0.03] |  |  |
| Religious denomination (1=Muslim) | 0.06 | [0.04] |  |  |
| Migrant background (1=both parents born abroad) | 0.03 | [0.03] |  |  |
| Employment (age 20) | -0.09** | [0.03] |  |  |
| University (age 20) | 0.05 | [0.04] |  |  |
| Achievement loss | 0.02 | [0.05] | -0.01 | [0.04] |
| Relational loss | -0.08* | [0.04] | 0.01 | [0.03] |
| Victimization | <0.01 | [0.06] | -0.02 | [0.05] |
| Contact with CJS | -0.09 | [0.07] | -0.02 | [0.06] |
| Social exclusion | 0.16*** | [0.03] | 0.06 | [0.03] |
| Low self-control | 0.27*** | [0.04] | 0.15** | [0.06] |
| Coping skills | -0.06* | [0.02] | -0.06* | [0.03] |
| Police legitimacy | -0.08** | [0.02] | 0.01 | [0.03] |
| Perceived peer disapproval of violence | -0.16*** | [0.03] | -0.16*** | [0.04] |
| Deviant behavior | 0.05*** | [0.01] | 0.04** | [0.01] |
| Wave (1=age 20) |  |  | -0.14*** | [0.02] |
| σ_u_ | 0.31 | | | |
| σ_e_ | 0.47 | | | |
| ICC (ρ) | 0.30 | | | |
| R^2^ overall | 0.27 | | | |
| R^2^ between | 0.33 | | | |
| R^2^ within | 0.15 | | | |
| Notes. Unstandardized coefficients are reported, with standard errors in brackets. ICC = interclass correlation. SE= standard error. Hybrid models estimate within-individual effects using random effects models. Estimates are based on 20 imputations, R^2^ values are combined using Fisher's z transformation and averaged over imputed data. *p<.05; **p<.01; ***p<.001. N (observations) = 2229. N (individuals) = 1117. | | | | |

The imputed results remain substantively the same compared to the listwise models, with two small exceptions. First, the between-individual coefficient for relational loss is significant in the imputed model (b=-0.08, p<.05). Second, the within-individual coefficient for deviant behavior is significant in the imputed model (b=0.04, p<.01). It is important to note that while the p-values differ between the imputed and listwise results, the size of the coefficients remains relatively similar between models (relational loss: b*_imputed_*=-0.08, b*_listwise_*=-0.07; deviant behavior: b*_imputed_*=0.04, b*_listwise_*=0.04).

*2. Results using alternative operationalizations of strain and significance loss*

Table A.2. Hybrid regression models using variety scales of adverse life events reflecting dimensions of strain and significance loss (ages 17 and 20)

|  | Between-individual effects | | Within-individual effects | |
| --- | --- | --- | --- | --- |
| Variables | b | SE | b | SE |
| Intercept | 1.56*** | [0.19] |  |  |
| Gender (1=male) | 0.19*** | [0.04] |  |  |
| Religious denomination (1=Muslim) | 0.08 | [0.04] |  |  |
| Migrant background (1=both parents born abroad) | 0.02 | [0.03] |  |  |
| Employment (age 20) | -0.06 | [0.03] |  |  |
| University (age 20) | 0.05 | [0.04] |  |  |
| Achievement loss | 0.01 | [0.03] | <0.01 | [0.03] |
| Relational loss | -0.07* | [0.03] | 0.02 | [0.03] |
| Victimization | -0.07 | [0.06] | -0.01 | [0.05] |
| Contact with CJS | -0.05 | [0.06] | -0.02 | [0.06] |
| Social exclusion | 0.16*** | [0.03] | 0.04 | [0.03] |
| Low self-control | 0.25*** | [0.05] | 0.19** | [0.06] |
| Coping skills | -0.06* | [0.02] | -0.06* | [0.03] |
| Police legitimacy | -0.10*** | [0.03] | <0.01 | [0.03] |
| Perceived peer disapproval of violence | -0.15*** | [0.03] | -0.14*** | [0.04] |
| Deviant behavior | 0.04** | [0.01] | 0.02 | [0.01] |
| Wave (1=age 20) |  |  | -0.15*** | [0.03] |
| σ_u_ |  | 0.29 |  |  |
| σ_e_ |  | 0.47 |  |  |
| ICC (ρ) |  | 0.29 |  |  |
| R^2^ overall |  | 0.25 |  |  |
| R^2^ between |  | 0.3 |  |  |
| R^2^ within |  | 0.14 |  |  |
| Notes. Unstandardized coefficients are reported, with standard errors in brackets. ICC = interclass correlation. SE= standard error. Hybrid models estimate within-individual effects using random effects models. *p<.05; **p<.01; ***p<.001. N (observations) = 1820. N (individuals) = 910. | | | | |

Table A.3. Hybrid regression models using a single variety scale of adverse life events reflecting overall strain and significance loss (ages 17 and 20)

|  | Between-individual effects | | Within-individual effects | |
| --- | --- | --- | --- | --- |
| Variables | b | SE | b | SE |
| Intercept | 1.56*** | [0.19] |  |  |
| Gender (1=male) | 0.20*** | [0.04] |  |  |
| Religious denomination (1=Muslim) | 0.08 | [0.04] |  |  |
| Migrant background (1=both parents born abroad) | 0.03 | [0.03] |  |  |
| Employment (age 20) | -0.07* | [0.03] |  |  |
| University (age 20) | 0.04 | [0.04] |  |  |
| Strain | -0.04* | [0.02] | <0.01 | [0.02] |
| Social exclusion | 0.16*** | [0.03] | 0.04 | [0.03] |
| Low self-control | 0.25*** | [0.05] | 0.20** | [0.06] |
| Coping skills | -0.06* | [0.02] | -0.06* | [0.03] |
| Police legitimacy | -0.10*** | [0.03] | <0.01 | [0.03] |
| Perceived peer disapproval of violence | -0.15*** | [0.03] | -0.14*** | [0.04] |
| Deviant behavior | 0.04** | [0.01] | 0.02 | [0.01] |
| Wave (age 20) |  |  | -0.14*** | [0.03] |
| σ_u_ |  | 0.29 |  |  |
| σ_e_ |  | 0.47 |  |  |
| ICC (ρ) |  | 0.29 |  |  |
| R^2^ overall |  | 0.25 |  |  |
| R^2^ between |  | 0.3 |  |  |
| R^2^ within |  | 0.14 |  |  |
| Notes. Unstandardized coefficients are reported, with standard errors in brackets. ICC = interclass correlation. SE= standard error. Hybrid models estimate within-individual effects using random effects models. *p<.05; **p<.01; ***p<.001. N (observations) = 1820. N (individuals) = 910. | | | | |

**References**

Azur, M.J., Stuart, E.A., Frangakis, C., & Leaf, P.J. (2011). Multiple imputations by chained equations: what is it and how does it work? International Journal in Methods in Psychiatric Research, 20, 40–49.

Gelman, A. & Hill, J. (2006). *Data Analysis Using Regression and Multi- level/Hierarchical Models*. Cambridge, U.K.: Cambridge University Press.

Rubin, D. (1987). *Multiple Imputation for Nonresponse in Surveys*. New York, NY: Wiley.
